# A comparative analysis of predictors of teenage pregnancy and its prevention in a rural town in Western Nigeria

**DOI:** 10.1186/1475-9276-11-37

**Published:** 2012-07-30

**Authors:** Olorunfemi E Amoran

**Affiliations:** 1 Department of Community Medicine and Primary Care, College of Health Sciences, Olabisi Onabanjo University Teaching Hospital, Sagamu, Nigeria

**Keywords:** Predictors, Teenage pregnancy, Prevention, Rural town, Nigeria

## Abstract

**Introduction:**

Teenagers younger than 15 are five times more likely to die during pregnancy or childbirth than women in their twenties and mortality rates for their infants are higher as well. This study was therefore designed to determine the recent prevalence and identify factors associated with teenage pregnancy in a rural town in Nigeria.

**Methods:**

This study is an analytical comparative cross-sectional study. A total sample of all pregnant women attending the primary health care in Sagamu local government area, Ogun State within a 2 months period were recruited into the study.

**Results:**

A total of 225 pregnant women were recruited into the study. The prevalence of teenage pregnancy was 22.9%. Teenagers [48.2%] reported more unwanted pregnancy when compared with the older age group [13.6%] [OR = 5.91, C.I = 2.83-12.43]. About half 33 [41.1%] of the teenage pregnant women and 28.6% of the older pregnant women did not know how to correctly use condom to prevent pregnancy [OR = 0.57, C.I = 0.29-1.13]. Predictors of teenage pregnancy were low social class (OR = 2.25, C.I = 1.31-3.85], Religion (OR = 0.44, C.I = 0.21-0.91], being a student (OR = 3.27, C.I = 1.02-10.46) and having a white collar job (OR = 0.09, C.I = 0.01-0.81).

**Conclusion:**

The study concludes that employment in an established organization (white collar job) is highly protective against teenage pregnancy while students are becoming increasingly prone to early pregnancy. Government should structure employment in low income countries in such a way as to give a quota to adolescents who are unable to continue their education.

## Introduction

Globally, young people aged between 15 and 24 years make up 1.2 billion of the world's population. The majority live in Sub-Saharan Africa and are vulnerable to teenage pregnancies and HIV infection [1]. Unwanted pregnancies and HIV infection continue to be daunting problems for young people, and studies indicate that HIV-infected youth face the greatest dilemmas [2,3]. Great attentions have been given to prevention of teenage pregnancy in recent times leading to several campaigns to prevent teenage pregnancy [4-6]. This is because it has been viewed as a negative phenomenon in modern times because of the attending negative effect on the health of these young teenagers. Despite the establishment of national teenage pregnancy programmes and strategies, [7] teenage birth rates have increased globally [8,9].

Teenage pregnancy might contribute to the cycle of poverty WHO [10]. In addition to the lost potentials of girls who are married off, there are real cost associated with women's health and infant mortality. The teenage pregnant girl is exposed to torture, abuse, and the risk of the deadly HIV/AIDS infection. Some young girls are forced into marriage at a very early age [11]. Others are simply too young to make an informed decision about their marriage partner or about the implications of the marriage itself. Early marriage deprives a girl of her adolescence [12]. Teenagers younger than 15 are five times more likely to die during pregnancy or childbirth than women in their twenties and mortality rates for their infants are higher as well. Recent studies have shown that associations among young men and women are largely explained by socioeconomic confounding [13-15].

A vast literature describing randomized, controlled trials clearly demonstrates that interventions with attention to specific elements can be successful in reducing and preventing sexual risk behaviours including unwanted pregnancy among teenagers [16-24]. However, available data indicate that intervention effects from such campaign wane over time, there is a paucity of information regarding long-term effects from prevention efforts and factors that may sustain such effects, particularly among preadolescents and adolescents as they mature and face changing personal and social environments [25-28]. Several social factors such as religious beliefs, idleness and economic factors have been identified as factors contributing to early pregnancy and marriage [29-34].

This study was therefore designed to determine the recent prevalence and identify factors associated with teenage pregnancy in a semi rural area in Nigeria. This has implications in the development of policies that will reduce the incidence of teenage pregnancy and give equal access to essential facilities which subsequently improve the health of the adolescents in low income countries such as Nigeria.

## Materials and methods

### Background of the study area

The study was conducted in Sagamu local government area (SLGA) Ogun state, which is located in the South Western part of Nigeria. Sagamu local government area is one of the 20 local government area in Ogun state. It was carved out of the former Ijebu Remo local government in 1991 and has a total land area of 68.03km^2^. It is bounded on the west by the Obafemi Owode local government area, on the east by both Ikenne and Odogbolu local government area and also shares a boundary with Ikorodu local government area of Lagos state in the south. According to the 2006 census, the area has a population of 253,412 inhabitants which consists of mainly remo-speaking people of Ogun state. Other ethnic groups like the Hausas, Igbos and the Benue people are well represented. Most of the towns are either semi-urban or rural. Other major towns in the local government besides Sagamu include Ogijo, Sotubo, Ode-lemo, Emuren and Simawa. The local government has 15 political wards, 12 of which fall within the Sagamu metropolis. This area is a major transit region between the southwest, southeast and the northern part of Nigeria.

There are seven centers for primary health care services and five other health posts spread all over the local government area. There are 52 registered birth attendants and one general and a teaching hospital. As at the time of this study, those primary health care centers that provide antenatal services are located at Ogijo, Sabo and Makun (the other primary health care centers were no longer functional, due to logistic reasons). Industries present in the local government area include the West African Portland Cement (WAPCO), Nulec industries, Sparkwest Nigeria Limited and branches of First bank, Guarantee trust bank, Wema bank and Zenith bank amongst others.

### Study design

This was an analytical cross-sectional study that quantitatively explored the factors associated with teenage pregnancy.

### Sampling size

The minimum sample size required for the study was estimated to be 138 using the formula

(1) $$n={\text{Z}}_{\alpha }^{2}\text{pq}/d^{2},$$

where n is the sample size,

Zα   is the standard normal deviate, set at 1.96 (for 95% confidence interval)

 d   is the desired degree of accuracy (taken as 0.050 and

 p   is the estimate of our target population having teenage pregnancy = 15%. [35].

(2) $$\begin{array}{c} N=1.96\text{X }1.96X0.15X0.85=195\\ 0.05^{2} \end{array}$$

Adjustment for a 10% rate of non-responses and invalid responses yielded a final sample size of 215.

### Sampling technique

Total sampling of all consenting pregnant women attending ANC at the three PHC offering ANC in the local government area for the first time were recruited during the 2 months study period.

### Data collection

Women who consented to take part in the study were interviewed using a structured questionnaire, which was administered by a trained interviewer. The interviewers were all female medical students rotating through the Community Medicine and primary health care department of the Olabisi Onabanjo University Teaching Hospital during the period of the study and one resident doctor. The data were collected on antenatal clinic days by the interviewers at the respective PHC centres. All consenting pregnant women were interviewed individually over a 10 to 15minute period in a language they can understand before they are given any health talk. Completed questionnaires were scrutinized on the spot and at the end of the daily field sessions for immediate correction of erroneous entry.

The instrument used was a structured questionnaire consisting of 2 parts, namely socio-demographic section (Section A), and pregnancy and sexual history (Section B). Section A includes information on socio-demographic data such as age, marital status, religion, employment status, ethnic group and educational status.

The questionnaire was pretested among 30 women in their first pregnancies receiving antenatal care at primary health care facilities in Ikenne local government, a nearby local government to the study area. Appropriate adjustments were then made to the questionnaire to improve its internal validity.

### Criteria for inclusion

• Subject must reside within Sagamu local government area (SLGA) of Ogun state.

• Subject must be in her first pregnancy and attending the PHC centre for the first time.

• Subject must not have received prior health talk

### Criteria for exclusion

• Subjects resident outside SLGA of Ogun state

• Subjects with disabilities that disallowed responses to questionnaire

• Subjects refusing to take part in the study

### Ethical consideration

Ethical clearance was obtained from the Olabisi Onabanjo Teaching Hospital Ethics Board. Confidentiality on candidate's information was maintained. Permission of the State Ministry of Health, HIV/AIDS Control Division were obtained before the commencement of the study.

At each of the selected study site, the matron and medical officer in-charge were informed for consent and antenatal clinic day(s) before the commencement of the study. The purpose, general content and nature of the study were explained to each respondent to obtain verbal and written consent before inclusion into the study. Informed consent was also obtained from the husbands or parents that followed the teenagers to the health facilities.

### Data analysis

The data were coded and entered into a computer database using SPSS 15 statistical software. Percentages or means and standard deviation were computed for baseline characteristics of women interviewed. The data analysis focused on univariate frequency table and bivariate cross tabulations that identify important relationships between variables. Respondents were categorized into low and high socioeconomic status using location of resident as cut off. Teenage Pregnancy was as defined by WHO as any pregnancy of a girl age 10 to 19.

The relationships between variables were examined through bivariate analysis, by computing odds ratio at 95% confidence level and chi squared and t-tests where appropriate. Predictor variables were restricted to outcome measures that were statistically significant. A p-value ≤ 0.05 or confidence limits which did not embrace unity (1) was considered as statistical significance.

## Results

### Socio-dermographic characteristics of respondents

A total of 225 pregnant women attending the ANC clinics of the primary health care centres for the first time within the study period were recruited into the study. The age of the respondents ranged from 14 to 40 years, (mean 24.34 ± 5.18 years). Majority 175 [77.8%] of the respondents were married with 62.7% being Christians and 37.3% Muslims. Three quarter 75.9% were of the Yoruba tribe, 10.7% were Hausas and 10.3% were Igbos and 3.1% were from other tribes. About half 49.1% of the respondents have completed a secondary education, 9.4% had a primary education and 8.5% had no education. Majority 82.7% of the respondents were either in training or employed. The prevalence of teenage pregnancy was 22.9%.

### Prevention of unwanted pregnancy

Teenagers [48.2%] reported more unwanted pregnancy when compared with the older age group [13.6%] [OR = 5.91, C.I = 2.83-12.43]. Almost all [99.2%] respondents reported use of condom during risky sexual intercourse. About half 33 [41.1%] of the teenage pregnant women and 28.6% of the older pregnant women did not know how to correctly use condom to prevent pregnancy and HIV/AIDS infection [OR = 0.57, C.I = 0.29-1.13]. Majority 195 (86.7%) of the respondents observe the fertile period to avoid unwanted pregnancy when involved with regular sexual partner. This practice is less common among the teenage respondents [OR = 0.65, C.I = 0.27-1.61]. Furthermore only 5.8% of the respondents have ever used control pills for the prevention of unwanted pregnancy. Table 1 shows the commonest methods of prevention of unwanted pregnancy among the respondents.

**Table 1:** Teenage Pregnancy by Prevention of Unwanted Pregnancy and other Social factors

	**Total No (%)**	**Teenage Pregnancy No (%)**	**Older Pregnant Women No [%]**	**Unadjusted Odds Ratio**
**Marital Status**				
Never Married	50 [22.2]	27 [48.2]	23 [13.6]	5.91 [2.83-12.43
Married	175 [77.8]	29 [51.8]	146 [86.4]	1.00
Total	225 [100.0]	56 [100.0]	169 [100.0]	
**Ethnicity**				
Yoruba	171 [75.6]	44 [78.6]	127 [75.0]	2.10 [0.24-47.47]
Igbo	24 [10.7]	3 [5.4]	21 [12.5]	0.86 [0.06-25.69]
Hausa	23 [10.3]	8 [14.3]	15 [8.9]	3.20 [0.27-83.54]
Others	8 [3.6]	1 [1.8]	6 [3.6]	1.00
**Level of Education**				
Nil	19 [8.5]	2 [3.6]	17 [10.1]	0.97 [0.13-5.71]
Primary	21 [9.4]	11 [19.4]	10 [6.0]	9.07 [2.58-33.07]
Secondary	110 [49.1]	35 [62.5]	75 [44.6]	3.85 [1.57-9.73]
Post -Secondary	74 [33.0]	8 [14.3]	66 [38.3]	1.00
**Correct Condom use**				
Correct use	153 [68.3]	33 [58.9]	120 [71.4]	0.57 [0.29-2.21]
Incorrect use	72 [31.7]	23 [41.1]	49 [28.6]	1.00
**Use of fertile period**				
Observe	194 [86.8]	46 [82.1]	148 [87.6]	0.65 [0.27-1.61]
Do not observe	31 [13.2]	10 [17.9]	21 [12.4]	1.00
**Use of Contraceptive pills**				
Ever use	13 [5.8]	4 [7.1]	9 [5.3]	1.37 [0.34-5.14]
Never use	212 [94.2]	52 [92.9]	160 [94.7]	1.00

### Factors associated with teenage pregnancy

Teenage pregnancy was statistically significantly associated with primary [OR = 9.07, C.I = 2.58-33.07] and secondary level of education [OR = 3.85, C.I = 1.57-9.73] when compared with post secondary education. Those from the low socio-economic background were about 4 times more likely to be pregnant as a teenager when compared to those from high socioeconomic background [OR = 3.81, C.I = 1.35-11.61]. Significantly more (58.9%) of the teenage pregnant women were Muslims (OR = 3.32, C.I = 1.70-6.52]. Among the ethnic groups the Hausas [34.8%] had the highest proportion of teenage pregnancy followed by the Yorubas [25.9%] OR = 3.20, C.I = 0.27-83.54]. Table 2 shows the relationship between socio-dermographic characteristics and teenage pregnancy.

**Table 2:** Social Characteristics Predictive of Teenage Pregnancy

	**Total**	**Teenage Pregnant Women**	**Matured Pregnant Women**	**Unadjusted Odds Ratio OR [95% C.I]**	**Adjusted Odds Ratio OR [95% C.I]**
**Religion**					
Christianity	141 [62.7]	23 [41.1]	118 [69.8]	1.00	0.44 [0.21-0.91]
Islam	84 [37.3]	33 [58.9]	51 [30.2]	3.32 [1.70-6.52]	1.00
**Occupation**					
Unemployed	39 [17.3]	11 [19.6]	28 [16.6]	1.00	1.00
White collar job	52 [23.1]	1 [1.8]	51 [30.2]	0.05 [0.00-0.41]	0.09 [0.01-0.81]
Traders	55 [24.4]	9 [16.1]	46 [27.2]	0.5 [0.16-1.50]	0.65 [0.21-2.00]
Students	48 [21.3]	20 [35.7]	28 [16.6]	1.82 [0.67-4.96]	3.27 [1.02-10.46]
Apprentices	31 [13.8]	15 [26.8]	16 [9.5]	2.39 [0.79-7.28]	2.01 [0.7-5.81]
**Social Class**					
Low	165 [73.3]	50 [89.3]	115 [68.0]	3.91 [1.49-10.84]	2.25 [1.31-3.85]
High	60 [26.7]	6 [10.7]	54 [32.0]	1.00	1.00

### Predictors of teenage pregnancy

Table 2 also shows the adjusted odds ratio for factors associated with teenage pregnancy among the study population. Low social class (OR = 2.25, C.I = 1.31-3.85], Religion (OR = 0.44, C.I = 0.21-0.91], being a student (OR = 3.27, C.I = 1.02-10.46) increased the probability of being pregnant while having a white collar job (OR = 0.09, C.I = 0.01-0.81) greatly reduce the probability of getting pregnant as a teenager after controlling for the effect of confounders.

## Discussion

The prevalence of teenage pregnancy in the study population was 22.9%. This finding is similar to others in Nigeria and other Africa countries and beyond [35-38]. This study suggests that despite several campaigns against teenage pregnancy in the developing countries, the incidence of teenage pregnancy has not decreased. Innovative approaches to promote the involvement of the teenagers in family planning activities are urgently needed. Rural Health care services should make clinics and family planning centres more youth-friendly, and enhance community mobilization and information-education-communication (IEC) activities to promote family planning among youths.

The study shows that those in low socioeconomic background are twice more likely to get pregnant as a teenager when compared with those from high socio-economic background. Sex education and sexual health services are not on their own effective strategies for encouraging teenagers to defer parenthood [39]; they need to be complemented by early childhood and youth development interventions that tackle social disadvantage [40]. Studies have also reported that economic deprivation is likely to influence risky sexual behaviours and heighten exposure to early pregnancy [41,42]. Early childhood interventions and youth development programmes provide enhanced educational and social support in the early years of life and engage young people in developing career aspirations. Social differences should be given special consideration in the design of programmes targeting adolescents in family life education.

This study implies that the teenage pregnant women compared to older women did not know how to correctly use condom to prevent pregnancy. This has also been reported in similar studies.[3,43,44] This indicates that the teenagers in the study population are deficient in the family life education essentially to provide them with greater negotiation skill and resisting power. Adolescents should be targeted in family life education programmes and health care services should be made more accessible and available to this younger population. This will help these teens access gynaecological care earlier in order to prevent early pregnancy.

The study shows that employment in an established organization (white collar job) is highly protective against teenage pregnancy while students are becoming increasingly prone to early pregnancy. Several studies have shown that dislike of school and a lack of opportunities for jobs and education have all emerged as explanatory factors in large scale national and international epidemiological analyses for causes of teenage pregnancy [45-47]. Government should structure employment in low income countries in such a way as to give a quota to adolescents who are unable to continue their education.

Religious belief is shown by the study to be an important predictor of teenage pregnancy. Religious beliefs form a conscious and integral part of the adolescents’ relationship. This study emphasises the role of religious beliefs in early pregnancy and marriage. This evidence has generated increased interest in the effects of interventions that religious beliefs are associated with teenage pregnancy [29-34]. Interventions sensitive to religious beliefs and ethnic peculiarities should be designed. Using religious leaders as vanguard of such campaign will be a very effective method to control teenage pregnancy.

Our study has certain limitations. The study findings are limited in terms of overall generalization and impact since it is not all pregnant women identified in Sagamu local government area actually deliver in these PHC clinics; most women deliver in other facilities or at home. Furthermore, applying the questionnaire in the health facility could lead to social desirability bias and the limitations of a cross-sectional study to explore risk and protective factors are important limitations of this study. Despite these limitations, we believe that our data provide useful information for the assessment of the prevalence of teenage pregnancy in Nigeria and identify factors associated with its perpetuation in Nigeria and other low income countries.

## Conclusion

The study concludes that employment in an established organization (white collar job) is highly protective against teenage pregnancy while students are becoming increasingly prone to early pregnancy. Government should structure employment in low income countries in such a way as to give a quota to adolescents who are unable to continue their education. Religion, job creation and social differences should be given special consideration in the design of programmes targeting adolescents in family life education.

## Competing interests

The author declare that he had no competing interests during the design up to the conduct of this study.

## Author’s contributions

AOE conceived the study, designed it and collected the data with the help of research assistants. He also analysed the data and drafted the manuscript.
